# Comparative Proteomic Analysis of Plasma Membrane Proteins in Rice Leaves Reveals a Vesicle Trafficking Network in Plant Immunity That Is Provoked by Blast Fungi

**DOI:** 10.3389/fpls.2022.853195

**Published:** 2022-04-25

**Authors:** Zhi Zhao, Meng Li, He Zhang, Yao Yu, Lu Ma, Wei Wang, Yunxin Fan, Ning Huang, Xinying Wang, Kunquan Liu, Shinan Dong, Haijuan Tang, Jianfei Wang, Hongsheng Zhang, Yongmei Bao

**Affiliations:** State Key Laboratory of Crop Genetics and Germplasm Enhancement, College of Agriculture, Jiangsu Collaborative Innovation Center for Modern Crop Production, Cyrus Tang Innovation Center for Crop Seed Industry, Jiangsu Province Engineering Research Center of Seed Industry Science and Technology, Nanjing Agricultural University, Nanjing, China

**Keywords:** *Oryza sativa* L., rice blast, plasma membrane, iTRAQ, proteomics, vesicle trafficking

## Abstract

Rice blast, caused by *Magnaporthe oryzae*, is one of the most devastating diseases in rice and can affect rice production worldwide. Rice plasma membrane (PM) proteins are crucial for rapidly and precisely establishing a defense response in plant immunity when rice and blast fungi interact. However, the plant-immunity-associated vesicle trafficking network mediated by PM proteins is poorly understood. In this study, to explore changes in PM proteins during *M. oryzae* infection, the PM proteome was analyzed *via* iTRAQ in the resistant rice landrace Heikezijing. A total of 831 differentially expressed proteins (DEPs) were identified, including 434 upregulated and 397 downregulated DEPs. In functional analyses, DEPs associated with vesicle trafficking were significantly enriched, including the “transport” term in a Gene Ontology enrichment analysis, the endocytosis and phagosome pathways in a Encyclopedia of Genes and Genomes analysis, and vesicle-associated proteins identified *via* a protein–protein interaction network analysis. OsNPSN13, a novel plant-specific soluble *N*-ethylmaleimide-sensitive factor attachment protein receptor (SNARE) 13 protein, was identified as an upregulated DEP, and transgenic plants overexpressing this gene showed enhanced blast resistance, while transgenic knockdown plants were more susceptible than wild-type plants. The changes in abundance and putative functions of 20 DEPs revealed a possible vesicle trafficking network in the *M. oryzae*-rice interaction. A comparative proteomic analysis of plasma membrane proteins in rice leaves revealed a plant-immunity-associated vesicle trafficking network that is provoked by blast fungi; these results provide new insights into rice resistance responses against rice blast fungi.

## Introduction

Rice blast, which is caused by *Magnaporthe oryzae* (*M. oryzae*), is one of the most devastating diseases in rice growing regions worldwide (Dean et al., 2005, 2012; Skamnioti and Gurr, 2009). To resist pathogen invasion, plants have developed a complex defense system that includes enemy molecule recognition, signal transduction and defensive responses throughout the immune system (Dodds and Rathjen, 2010; Andersen et al., 2018). In the first layer of plant innate immunity, pattern recognition receptors (PRRs) on the plasma membrane (PM) can detect pathogen-associated molecular patterns (PAMPs) and activate pattern-triggered immunity (PTI), which leads to the activation of signaling cascades by mitogen-activated protein kinases (MAPKs) and calcium-dependent protein kinases (CPKs), the transcriptomic reprogramming of defense-related genes, a burst of Ca^2+^ and reactive oxygen species (ROS) and the deposition of callose (Chisholm et al., 2006; Jones and Dangl, 2006; Boller and Felix, 2009; Gu et al., 2017). However, adapted pathogens have evolved and release various effector proteins that cause effector-triggered susceptibility (ETS), which drives the evolution of the second layer of innate immunity in plants, termed effector-triggered immunity (ETI): ETI includes the sensing of effectors by nuclear-binding leucine-rich repeat receptors (NLRs); the hypersensitive response, especially programmed cell death (PCD); and the regulation of phytohormones (Durrant and Dong, 2004; Jones and Dangl, 2006; Dodds and Rathjen, 2010; Macho and Zipfel, 2014).

The PM acts as the primary plant-microbe interaction interface during pathogen infection. Due to its physiochemical characteristics of heterogeneity, hydrophobicity and low abundance, the PM is often underrepresented at an early stage (Marmagne et al., 2004). In recent years, diverse proteomic technologies have been widely used in plants, including one- or two-dimensional electrophoresis and liquid chromatography (LC) to separate membrane proteins, and gel-based and mass spectrometry (MS)-based (label or label-free) methods for the quantification of membrane proteins; and many proteins have been shown to be related to defense responses (Benschop et al., 2007; Komatsu, 2008; Kota and Goshe, 2011; Hopff et al., 2013; Yadeta et al., 2013; Aloui et al., 2018; Witzel et al., 2018; Wei et al., 2020). Quantitative PM proteomics have shown that in *Arabidopsis*, after stimulation with the flagellin peptide flg22, transporters and receptor-like kinases (RLKs), especially the flagellin receptor FLS2, change significantly, while other proteins, including H^+^-ATPase, Ca^2+^-ATPase and V-ATPase, also change, accompanied by the rapid phosphorylation of the ABC transporter PDR8/PEN3 and the NADPH oxidase RbohD as immediate responses at the early defense stage (Nühse et al., 2007; Keinath et al., 2010); after the activation of ETI, dynamic signaling events, primary and secondary metabolism, vesicle trafficking and transport change significantly (Elmore et al., 2012). In rice suspension cells expressing the disease resistance gene *Xa21*, H^+^-ATPases, phosphatases, hypersensitive-induced response protein (OsHIR1), prohibitin (OsPHB2), zinc finger proteins, universal stress proteins (USPs) and heat shock proteins are differentially regulated at the early defense stage after inoculation with bacterial blight (*Xanthomonas oryzae* pv. *oryzae*) (Chen et al., 2007). Recently, isobaric tags for relative and absolute quantification (iTRAQ) have been widely used in proteomic analysis due to their high reliability and efficiency (Karp et al., 2010; Evans et al., 2012). However, the changes in the PM proteome in rice in response to rice blast fungus are still unknown.

Notably, in plants, vesicle trafficking is a key activity at the PM in the face of pathogen attacks. The delivery of defense proteins and antimicrobial substances to the extracellular space, the deposition of callose at the cell wall and the movement of PRRs to the PM are mediated by secretory-associated membrane trafficking (Wang et al., 2015); the recycling of ligand-free and ligand-bound PRRs, the delivery of pathogen effectors and transport toward vacuoles for degradation are mediated by endocytosis-associated membrane trafficking at the location of the host-pathogen interaction (Kale and Tyler, 2011; Gu et al., 2017). Diverse proteins participate in the vesicle trafficking network at the PM, including in the process of fusion between vesicles and the PM in the secretory system and the formation of vesicles in endocytosis (Leborgne-Castel and Bouhidel, 2014). In the secretory pathway, the initial contact and recognition between vesicles and the PM are mediated by vesicle tethering factors, including the exocyst complex (SEC3, SEC5, SEC6, SEC8, SEC10, SEC15, EXO70, and EXO84) (Yu and Hughson, 2010; Žárský et al., 2013). After recognition, membrane fusion at the PM is mainly accomplished by soluble *N*-ethylmaleimide-sensitive factor attachment protein receptors (SNAREs) (Lipka et al., 2007).

The SNARE complex is assembled by Q-SNAREs (Qa, Qb and Qc-SNAREs) on the target membrane and an R-SNARE on the vesicle membrane and leads to membrane fusion (Fasshauer et al., 1998; Sanderfoot et al., 2000; Bassham and Blatt, 2008). Syntaxin of plants (SYP) is a Q-SNARE and participates in various defense responses. AtSYP121 in *Arabidopsis* belongs to the SYP1 subfamily; it forms a ternary complex with AtSNAP33 (synaptosome-associated protein of 33 kDa, a SNAP25-type protein with Qb + Qc-SNARE domains) and AtVAMP721/722 (vesicle-associated membrane protein 721/722, an R-SNARE) to deliver defense components to point of the PM where the fungus is attacking the extracellular space (Collins et al., 2003; Kwon et al., 2008). OsSYP121 in rice accumulates at the sites of rice blast fungus penetration and forms a ternary complex with the SNAP25-type proteins OsSNAP32 and OsVAMP714/724 (Bao et al., 2008b; Cao et al., 2019). SYP121/ROR2 in barley (*Hordeum vulgare*) forms a binary SNARE complex with HvSNAP34 and accumulates focally near the papilla to fight against powdery mildew penetration (Assaad et al., 2004; Bhat et al., 2005). SYP122 functions redundantly with its homolog SYP121 and resists penetration by facilitating cell wall deposition in *Arabidopsis* (Nühse et al., 2003; Assaad et al., 2004; Zhang et al., 2007). SYP123 in *Arabidopsis* contributes to induced systemic resistance (ISR) and the establishment of plant defense responses (Rodriguez-Furlán et al., 2016). NbSYP132 in *Nicotiana benthamiana* engages in the immune response to the bacterial pathogen *Pseudomonas syringae* by secreting vesicles containing pathogenesis-related (PR) proteins (Kalde et al., 2007). The SYP4-group proteins SYP41/42/43, located at the *trans*-Golgi network/early endosome (TGN/EE) in *Arabidopsis*, are crucial to extracellular resistance by regulating the dynamic relocation of VAMP721 to sites of plant–fungus interaction and facilitating the formation of the SYP121/VAMP721 SNARE complex (Uemura et al., 2012, 2019). The SYP7 group is a plant-specific Qc-SNARE subfamily, and transgenic plants overexpressing *OsSYP71* show enhanced resistance to rice blast, while silencing *SYP71* in wheat results in susceptibility to stripe rust disease (Bao et al., 2012; Liu et al., 2016). The AtBET12 Qc-SNARE interacts with the AtMEMB12 Qb-SNARE and engages in endoplasmic reticulum (ER)-to-Golgi anterograde protein trafficking, whereas it plays a repressive role in PR1 secretion (Zhang et al., 2011; Chung et al., 2018). Rab is the largest family of small GTPases, and they work with SNARE proteins to promote membrane fusion and regulate the process of secretion-dependent immune activation (Yang, 2002; Uemura et al., 2004). In plants, the Rab family can be divided into eight subfamilies, including Rab1, Rab2, Rab5, Rab6, Rab7, Rab8, Rab11, and Rab18 (Agarwal et al., 2009). RabA1b regulates the transport of the PRR Flagellin-Sensitive 2 (FLS2) from the TGN/EE to the PM (Choi et al., 2013); RabA4c contributes to the increase of callose deposition at the penetration sites of the powdery mildew fungal pathogen *Golovinomyces cichoracearum* (Ellinger et al., 2014); and RabE1d in *Arabidopsis* facilitates the secretion of PR1 and enhances resistance against *Pst* DC3000 (Speth et al., 2009). However, few PM proteins that participate in vesicle trafficking against *M. oryzae* infection in rice have been reported, and the changes that occur in the PM protein network during *M. oryzae* infection remain largely unknown.

In this study, the iTRAQ method was used to identify the changes in the PM proteome in the highly resistant rice landrace Heikezijing (Hei) after 24 h of *M. oryzae* infection. A total of 2,970 proteins were identified, 831 of which were responsive in resistance against *M. oryzae.* Gene Ontology (GO) enrichment analysis, Kyoto Encyclopedia of Genes and Genomes (KEGG) pathway analysis and a protein interaction network showed that many proteins were involved in vesicle trafficking and had obvious functional tendencies toward transport, endocytosis and phagosomes. Ten DEPs were validated at the transcript level, and a SNARE protein named NPSN (novel plant-specific SNARE) 13 actively responded to *M. oryzae* infection, contributed to rice blast resistance and was mainly located at the PM. Our study sheds light on some of the significant roles of PM proteins and vesicle proteins in *M. oryzae*–rice interactions.

## Materials and Methods

### Plant Materials and Growth Conditions

The rice (*Oryza sativa* L. *japonica*) landraces Hei and Su, which are resistant and susceptible, respectively, to *M. oryzae* strain *Hoku1*, along with three transgenic overexpression lines and two transgenic knockdown lines of *OsNPSN13* were used in this study. Rice seeds were sown in pots containing 75% garden soil and 25% nutrient soil and grown in a greenhouse (16 h light and 8 h dark for cycle at 25°C ± 3°C) for 3 weeks for rice blast fungus inoculation.

### Pathogen Inoculation and Disease Evaluation

The blast strain *Hoku1* was used for blast fungus inoculation in this study. Three-week-old rice seedlings were inoculated by spraying them with a spore suspension (1 × 10^–5^ spores mL^–1^ in 0.025% [w/v] Tween 20) as previously reported (Wang et al., 2002). The inoculated seedlings were kept for 24 h in a dark incubation room with 100% relative humidity at 26°C and then moved to the greenhouse for disease development. Disease evaluation, including lesion number and lesion length, was conducted 7 days after inoculation.

### Extraction and Purification of Plasma Membrane Proteins

**FIGURE 1:**
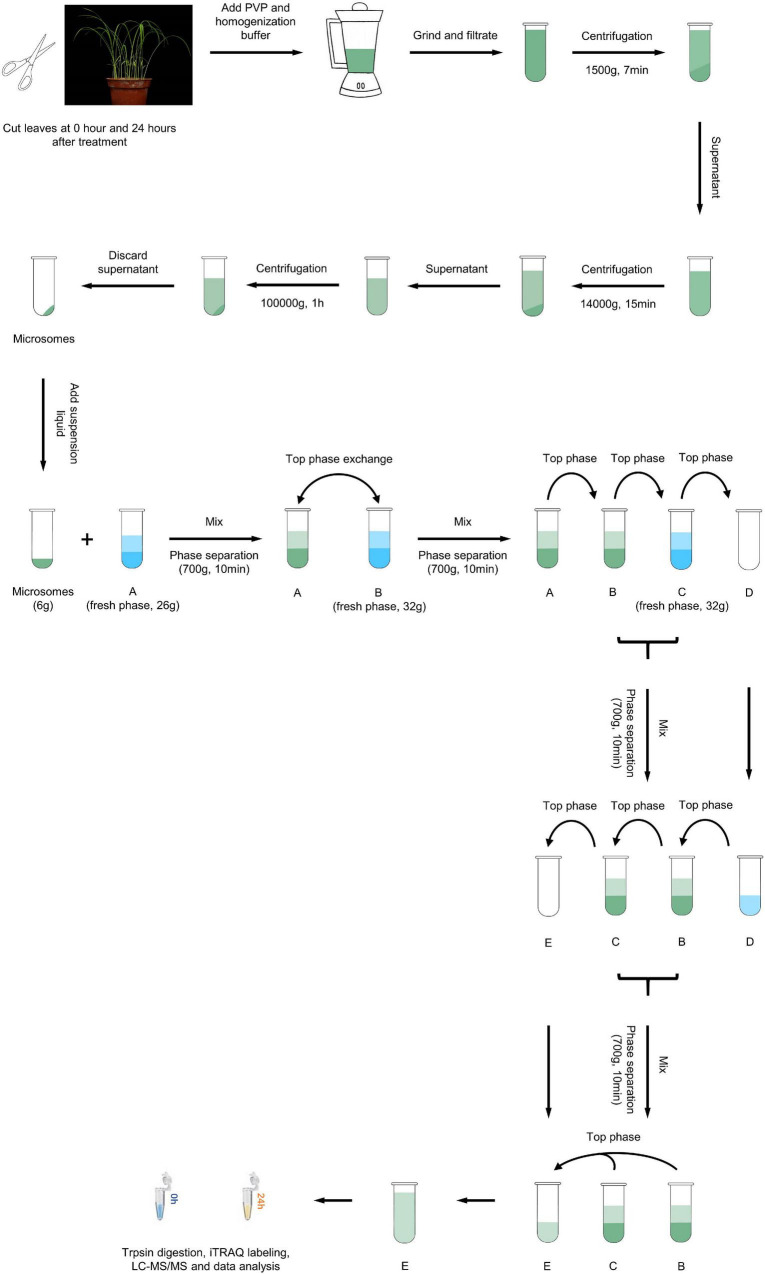
Overview of the experimental scheme. Schematic representation of the proteomics workflow. Leaf samples were collected at 0 and 24 h after *M. oryzae* infection from the resistant rice landrace Hei. Total PM protein was extracted, digested and quantified *via* iTRAQ labeling and LC–MS/MS analysis. Bioinformatic analyses were used to explore the constitution and function of DEPs.

Rice leaves that were either non-inoculated or had been inoculated for 24 h were sampled, flash frozen in liquid nitrogen and stored at –80°C for PM protein extraction. PM protein extraction and purification were performed according to the method of Cheng et al. (2009). The leaf samples were cut and ground in ice-cold homogenizing buffer containing 250 mM sucrose, 2 mM EGTA, 10% v/v glycerol, 0.5% w/v BSA, 2 mM DTT, 1 mM PMSF, 1.5% PVP, 5 mM β-mercaptoethanol, and 50 mM 1,3-bis [Tris(hydroxymethyl)-methylamino] propane (BTP) and adjusted to pH 7.8 with MES. The homogenate was filtered twice through two layers of cheesecloth and then centrifuged at 1,500 × *g* for 7 min and 10,000 × *g* for 15 min to remove debris. The supernatants were centrifuged further at 100,000 × *g* for 1 h. The microsomal pellets were resuspended in phase buffer (250 mM sucrose, 3 mM KCl, and 5 mM KH_2_PO_4_, pH 7.8) and then fractionated *via* two-phase partitioning in aqueous dextran T500 and PEG (6.3% w/v PEG 3350, 6.3% w/v dextran, 0.25 M sucrose, dissolved in 10 mM KH_2_PO_4_/K_2_HPO_4_ buffer, 35 mM NaCl, pH 7.8). The upper phase, which contained the PM vesicles, was collected and repartitioned three times against a fresh lower phase (Figure 1). The final upper phase was diluted with phase buffer and centrifuged at 160,000 × *g* for 40 min. The PM pellets were washed twice with resuspension buffer (250 mM sucrose, 3 mM KCl, and 5 mM BTP/MES, pH 7.8), centrifuged at 160,000 × *g* for 1 h, resuspended in 600 μl resuspension buffer, and stored at –80°C before use.

Each sample was added to 30 μl of SDT buffer (4% SDS, 1 mM DTT, 150 mM Tris-HCl pH 8.0) and incubated for 5 min, then disrupted with an ultrasonic disruptor (80 W, 10 s disruption with 15 s intermission repeated 10 times), incubated for 5 min, and centrifuged at 14000 × *g* for 15 min; then, the supernatant was collected. Samples were qualified using a bicinchoninic acid (BCA) protein assay.

### Protein Digestion and Isobaric Tags for Relative and Absolute Quantification Labeling

One mixture containing samples from all groups was used as a control and is indicated as REF. Before the iTRAQ labeling experiments, 200 μg of each sample had DTT (dithiothreitol) added to 100 mM, followed by incubation for 5 min; then, 100 μl of UA buffer (8 M urea, 150 mM Tris-HCl pH 8.0) was added, the sample was centrifuged at 14,000 × *g* for 15 min, and the filtrate was discarded. The cells were alkylated with 100 μl IAA (iodoacetamide) (50 mM IAA in UA) for 30 min in the dark at room temperature. For trypsin digestion, 40 μl of trypsin buffer (4 μg of trypsin in 40 μl of dissolution buffer) was added and digested at 37°C for 16–18 h. iTRAQ labeling was performed using an iTRAQ Reagent-8plex Multiplex Kit (AB SCIEX) according to the manufacturer’s protocol. iTRAQ labeling was performed as in Supplementary Table 1: REFs were labeled with iTRAQ reagent 113, the three biological replicates at 0 h were labeled with iTRAQ reagents 114, 115, and 116, and the three biological replicates at 24 h were labeled with iTRAQ reagents 117, 118, and 119.

### Strong Cation Exchange Chromatography Fractionation

The peptide mixture was fractionated on an AKTA Purifier 100 (GE Healthcare, Chicago, IL, United States) equipped with a 4.6 × 100 mm polysulfoethyl column (PolyLC Inc., Columbia, MD, United States). The Strong Cation Exchange (SCX) gradient was 100% buffer A (10 mM KH_2_PO_4_ pH 3.0, 25% ACN) for 30 min, 90% buffer A and 10% buffer B (10 mM KH_2_PO_4_ pH 3.0, 500 mM KCl, 25% ACN), 80% buffer A and 20% buffer B, 55% buffer A and 45% buffer B, 100% buffer B for 10 min, and 100% buffer A for 15 min. The flow rate was 1000 μl/min. Fractions were collected every minute during the run. Subsequently, all 30 collected fractions were mixed into 10 components, lyophilized, and kept at –80°C before high-performance liquid chromatography-tandem mass spectrometry (HPLC–MS/MS) analysis.

### HPLC–MS/MS Analysis

The peptides were separated on an Easy nLC system with a Thermo Scientific EASY analytical column (75 μm × 100 mm 3 μm-C18), which was interfaced with a Q-Exactive mass spectrometer (Thermo Finnigan, San Francisco, CA, United States). Mobile phase A was 0.1% formic acid, and mobile phase B consisted of 100% acetonitrile and 0.1% formic acid. Peptides were separated at a flow rate of 250 nl/min with the following gradients: 0–35% phase B, 100 min; 35–100% phase B, 8 min; and 100% phase B for 12 min. There was a full-scan mass spectrum in the Orbitrap (300–1800 m/z, 70,000 resolution) with an automatic gain control (AGC) target value of 3e6. A data-dependent acquisition method was used to collect the generated MS/MS spectra at a resolution of 17,500 with an AGC target of 1e5 and a maximum injection time (IT) of 60 ms for the top 10 ions observed in each mass spectrum. The isolation window was set at 2 m/z, the dynamic exclusion time was 40 s, and the normalized collisional energy (NCE) was set at 30 eV.

The raw data were processed using Mascot2.2 and Proteome Discoverer1.3 software (Thermo Fisher Scientific, Waltham, MA, United States). The mass spectra of the raw data were searched against the UniProt_Oryza.fasta database (144512 sequences, released February 2013). Trypsin was specified as a cleavage enzyme, allowing a maximum of 2 missing cleavages. Carbamidomethyl on cysteine was distinguished as a fixed modification. Oxidation on methionine and iTRAQ8plex (Y) were specified as variable modifications. The peptide mass tolerance was programmed to 20 ppm, and the fragment mass tolerance was programmed to 0.1 Da. The threshold score was set to 20, and the false discovery rate (FDR) threshold was adjusted to 0.01 (Sandberg et al., 2012). The quantitative analysis parameters of Proteome Discover1.3 are listed in Supplementary Table 2.

### Bioinformatics Analysis

For three biological replicates, the average ratio between the protein expression level at 24 h and the protein expression level before inoculation was defined as the fold change. A 1.1-fold or 0.909-fold change as the threshold with FDR < 0.1 (*p* value adjusted using the Benjamini–Hochberg method for multiple comparisons) was classified as a significant change (Benedikter et al., 2019). Volcano maps and heatmaps were made using the R package ggplot2 and TBtools software (Wickham, 2016; Chen et al., 2020). The functional annotation of DEPs was mapped with GO terms^1^ using the UniProt database, and the enrichment was calculated using singular enrichment analysis (SEA) in agriGO with the FDR < 0.05 (Du et al., 2010). The DEPs were further functionally analyzed using the KEGG database^2^ and the R package clusterProfiler (Yu et al., 2012). The protein--protein interaction (PPI) network was analyzed based on the STRING database^3^. The PPI network was obtained by setting the confidence value at 0.4, and then Cytoscape 3.6.0 was used to adjust the PPI map (Paul et al., 2003). DEPs were mapped to a pathway enrichment in biotic stress by using MapMan software (Thimm et al., 2004).

### qRT–PCR Analysis

The leaves of the resistant cultivar Hei were sampled at 0, 8, 24, 48, and 72 h after *M. oryzae* inoculation, frozen in liquid nitrogen and stored at –80°C. Total RNA was extracted using a universal plant total RNA extraction kit (Bioteke, Beijing, China). cDNA was synthesized using a HiScript II Q RT SuperMix kit (Vazyme, Nanjing, Jiangsu, China) for real-time quantitative PCR (qRT-PCR), which was performed using SYBR Green Master Mix (Vazyme, Nanjing, Jiangsu, China) according to the manufacturer’s protocol. The *actin* gene was used as an internal control, and the expression levels of genes were measured using the 2^–ΔΔ*CT*^ (cycle threshold) method (Pfaffl, 2001).

### Generation and Identification of Transgenic Plants

Full-length and reversed full-length *OsNPSN13* were inserted into the pCAMBIA1304S vector to generate the overexpression transgenic vector pCAMBIA1304S-*OsNPSN13* and the antisense transgenic vector pCAMBIA1304S-*rOsNPSN13*, respectively. These vectors were transformed into the susceptible rice landrace Suyunuo using the *Agrobacterium tumefaciens*-mediated method (Toki et al., 2006). Total DNA from leaves of different transgenic lines was extracted and used to identify overexpression and antisense transgenic plants *via* PCR amplification. The primers used are in Supplementary Table 3.

### Subcellular Localization of *OsNPSN13* in Protoplasts

The full-length *OsNPSN13* cDNA was amplified from Heikezijing and cloned into the pAN580 vector with the C-terminus of the fragment in frame with GFP. Protoplasts were extracted from young Heikezijing seedlings, and transient transformation with the plasmid was performed as described by Chen et al. (2006). 10 μg of plasmid DNA was added to 200 μL of suspended protoplasts (1 × 10^6^ cells mL^–1^) and incubated in the dark at 28°C. OsAMT3.2-RFP was used as PM marker. After 12 h, the transformed protoplast cells were observed using a Leica SP8 laser confocal microscope.

## Results

### Use of Isobaric Tags for Relative and Absolute Quantification to Quantitatively Identify Plasma Membrane Proteins That Respond to *Magnaporthe oryzae* Infection

**FIGURE 2:**
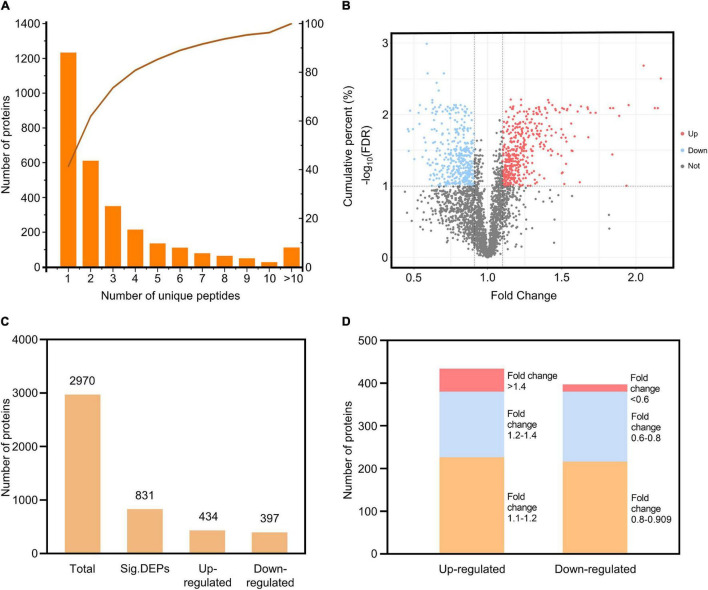
Quantitative identification of proteins from the iTRAQ analysis. **(A)** Distribution of the number of unique peptides for the identified proteins. The *X*-axis is the scope of the unique peptides, the left *Y*-axis is the number of proteins, and the right *Y*-axis is the cumulative percent. **(B)** Volcano plot of the proteins identified. The *X*-axis specifies the fold change between 24 h/0 h, and the *Y*-axis specifies the -log10 (FDR); upregulated, downregulated and non-significant proteins are shown as red, blue, and gray dots, respectively; gray vertical and horizontal lines reflect the filtering criteria (fold change > 1.1 or < 0.909 and FDR < 0.1). **(C)** The number of total proteins and DEPs (including upregulated and downregulated). **(D)** The distribution of DEP fold changes.

To investigate the roles of rice PM proteins against *M. oryzae* infection, we used iTRAQ to analyze the PM proteome in the resistant rice landrace Heikezijing (Hei). PM proteins were extracted from the leaves of rice seedlings and purified *via* two-phase partitioning in aqueous dextran T500 and PEG according to Cheng et al.’s (2009) method, then digested and labeled with iTRAQ reagents and quantitatively identified using LC–MS/MS (Figure 1). A total of 10608 unique peptides and 2,987 proteins were identified *via* the UniProt database (Supplementary Tables 4–6). The number of proteins in the three biological replicates of this study were 2982, 2986, and 2981 (Supplementary Figure 1), and there were 2978 common proteins in three repeats. For most proteins, fewer than 10 unique peptides were identified (Figure 2A). A total of 1,745 proteins contained at least 2 unique peptides, accounting for 58.62% of the total protein. The average protein identification coverage was 11.73%.

Based on the thresholds of FDR < 0.1 and fold change > 1.1 for upregulated DEPs and fold change < 0.909 for downregulated DEPs, 831 differentially expressed proteins (DEPs) were identified, including 434 upregulated DEPs and 397 downregulated DEPs (Figures 2B,C and Supplementary Tables 7, 8). Fifty-four upregulated DEPs had a fold change greater than 1.4, and 17 downregulated DEPs had a fold change less than 0.6 (Figure 2D). The distribution of fold changes showed that the PM proteome changed at different levels during the immune response against rice blast. The number of upregulated DEPs was approximately equal to the number of downregulated DEPs, and the three replicates for each treatment had good repeatability (Supplementary Figure 2). A total of 27.98% of all identified proteins changed during *M. oryzae* infection, suggesting that many PM proteins responded to and were involved in the plant defense process.

### Functional Characterization of Differentially Expressed Proteins

**FIGURE 3:**
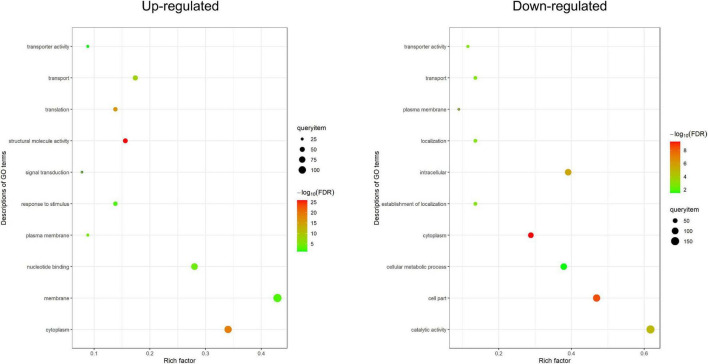
Gene ontology enrichment analysis. GO enrichment analysis of DEPs (FDR < 0.05). The enriched terms from three levels (biological process, molecular function, and cellular component) are shown in the figure. The *X*-axis is the rich factor, and the *Y*-axis is the description of GO term.

Based on Gene Ontology (GO) annotations, 434 up-regulated DEPs and 397 down-regulated DEPs were separately categorized into three major enrichment categories: biological process, cellular component and molecular function (FDR < 0.05). The results showed that 282 up-regulated DEPs (65%) were annotated at 10 levels, including four biological processes, three cellular components, and three molecular functions; 243 down-regulated DEPs (61%) were annotated at 10 levels, including four biological processes, four cellular components, and two molecular functions (Figure 3 and Supplementary Table 9). In the biological process category, up-regulated DEPs were enriched in translation (GO:0006412), transport (GO:0006810), signal transduction (GO:0007165) and response to stimulus (GO:0050896); down-regulated DEPs were enriched in transport (GO:0006810), establishment of localization (GO:0051234), localization (GO:0051179) and cellular metabolic process (GO:0044237). Intriguingly, many DEPs were related to vesicle trafficking, including 82 DEPs (49 up-regulated and 33 down-regulated) involved in transport, which implied that the trafficking system was activated by rice blast infection. In the cellular component category, up-regulated DEPs were enriched in membrane (GO:0016020), cytoplasm (GO:0005737) and plasma membrane (GO:0005886); down-regulated DEPs were enriched in cytoplasm (GO:0005737), cell part (GO:0044464), intracellular (GO:0005622) and plasma membrane (GO:0005886). At the level of molecular function, up-regulated DEPs were associated with structural molecule activity (GO:0005198), nucleotide binding (GO:0000166) and transporter activity (GO:0005215); down-regulated DEPs were associated with catalytic activity (GO:0003824) and transporter activity (GO:0005215). Both up-regulated and down-regulated DEPs were linked with transporter activity. Overall, based on GO enrichment analysis, the functional categories of the DEPs from the three different levels all pointed to the primary conclusion that vesicle trafficking played an important role in rice resistance against *M. oryzae*.

**FIGURE 4:**
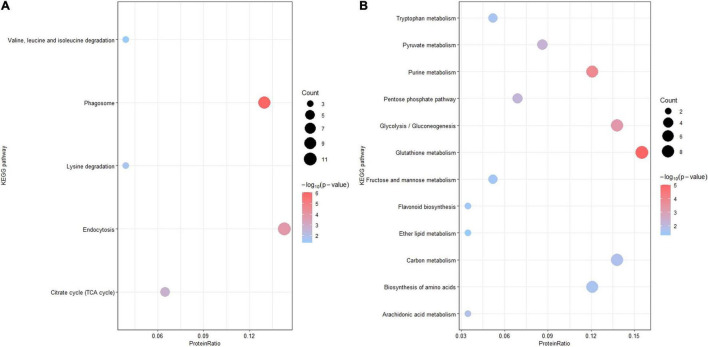
Kyoto Encyclopedia of Genes and Genomes pathway enrichment analysis. KEGG pathway enrichment analysis of DEPs (*p-*value < 0.05). **(A)** KEGG pathway enrichment analysis of upregulated DEPs. **(B)** KEGG pathway analysis of downregulated DEPs. The *X*-axis is the protein ratio (the number of DEPs annotated with this pathway term/the total number of DEPs), the *Y*-axis is the KEGG pathway, the size of the bubble represents the protein count, and the color of the bubble represents –log10 (*p-*value).

For further functional grouping, Kyoto Encyclopedia of Genes and Genomes (KEGG) pathway analysis was performed (Figure 4). Upregulated DEPs were generally assigned to the phagosome (osa04145), endocytosis (osa04144), the citrate cycle (osa00020), lysine degradation (osa00310) and valine/leucine/isoleucine degradation (osa00280); downregulated DEPs were mainly assigned to glutathione metabolism (osa00480), purine metabolism (osa00230), glycolysis (osa00010), pyruvate metabolism (osa00620) and the pentose phosphate pathway (osa00030). The top two most prominent enriched upregulated pathways were the phagosome and endocytosis, suggesting that vesicle trafficking is a crucial process in the resistance response (Table 1 and Supplementary Figure 3). The GO enrichment and KEGG pathway analyses jointly showed that multiple DEPs were engaged in vesicle trafficking, which suggested that we should analyze the detailed components and functions of this process.

**TABLE 1:** The list of up-regulated DEPs involved in phagosome and endocytosis according to KEGG enrichment analysis.

No.	Uniprot ID	Annotation	Average fold change
**Phagosome**			
1	Q40665	Tubulin beta-3 chain	1.12
2	Q10P12	V-type proton ATPase subunit a	1.12
3	Q8RU33	Probable V-type proton ATPase subunit d	1.15
4	Q6ZHA3	Rac-like GTP-binding protein 6	1.17
5	Q7XV86	Calnexin	1.17
6	Q6Z808	Rac-like GTP-binding protein 3	1.21
7	Q5JLU1	Os01g0714900 protein	1.22
8	Q8H7U1	Tubulin beta-2 chain	1.33
9	Q76FS3	Tubulin beta-6 chain	1.48
10	Q43594	Tubulin beta-1 chain	2.17
**Endocytosis**			
1	Q6AUF8	Os05g0564400 protein	1.11
2	Q943K7	70 kDa heat shock protein	1.12
3	Q2R0 × 0	Os11g0629200 protein	1.16
4	Q654U5	Dynamin GTPase	1.16
5	Q5JLU1	Os01g0714900 protein	1.22
6	Q75M17	Os05g0105100 protein	1.27
7	Q6ETY0	Vacuolar protein sorting-associated protein 29	1.30
8	Q6L502	Os05g0461300 protein	1.31
9	Q94J09	GTP-binding protein GTP1	1.33
10	Q84TA8	GTPBP	1.40

Of the 831 DEPs, 496 could be mapped in MapMan, and 136 of those were associated with biotic stress (Supplementary Figure 4). Forty-seven DEPs associated with signaling were the most enriched group, including 34 upregulated DEPs and 13 downregulated DEPs. Other physiological activities, including respiratory burst, hormone signaling and proteolysis, were also significantly changed. Diverse types of proteins were identified, including peroxidases, glutathione-S-transferases, transcription factors, beta-glucanases, heat shock proteins and secondary metabolites. The various DEPs engaged in rice blast resistance at the PM suggest that there must be an intricate and dynamic network linked with various biological processes at the PM during the plant immune response and during plant–pathogen interactions.

### A Protein–Protein Interaction Network of Selected Differentially Expressed Proteins Based on the STRING Database

**FIGURE 5:**
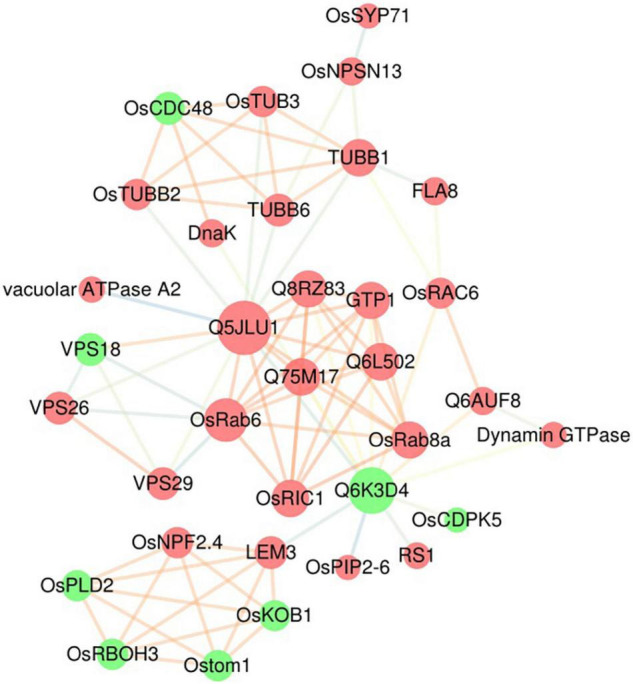
Protein–protein interaction network of DEPs. A protein–protein interaction network of DEPs associated with vesicle trafficking (including the transport terms from the GO analysis and the phagosome and endocytosis terms from the KEGG analysis) was built using the STRING database. Identified interactions with confidence scores ≥ 0.4 were visualized using Cytoscape software. The red nodes are upregulated DEPs, and the green nodes are downregulated DEPs; the size of the nodes represents the number of related interactions in the network; and the colors of the edges indicate the strength of the interaction: orange represents a strong interaction, and blue represents a weak interaction.

To decipher the functional networks, we used the STRING database to construct a PPI network of DEPs and obtain a putative association network. The results of the functional analysis showed that many DEPs participated in vesicle trafficking, so we focused on these DEPs, which encompassed one major GO enrichment term (transport) and two major pathways from the KEGG analysis (phagosome and endocytosis), to further understand their functions. The maximum confidence (score) cutoff was adjusted to 0.40, and the result was visualized using Cytoscape (Figure 5). The results showed that the most closely interacting DEPs (>9 interaction degrees) were found between multiple small GTPase subfamily proteins, including the Ras protein OsRIC1 (P40392) and unclassified Ras-related proteins (Q5JLU1, Q8RZ83, Q75M17, Q6AUF8, Q6L502), the Rab proteins OsRab6 (Q8H4Q9) and OsRab8a (Q84TA8), and the GTP-binding protein GTP1 (Q94J09); other types of proteins, including the vacuolar protein sorting-associated proteins VPS18 (Q6ZKF1), VPS26 (Q2R0X0) and VPS29 (Q6ETY0), a serine/threonine phosphatase (Q6K3D4), tubulin beta-1 chain protein TuBB1 (Q43594) and tubulin beta-6 chain protein TuBB6 (Q76FS3), also had close interactions. Thirteen of these 15 proteins were upregulated, which meant that active transportation was essential in rice blast resistance. Nine proteins were small GTPase-related transporting and vacuolar protein sorting-associated proteins, indicating that different types of vesicles and organelles participated in plant immunity.

### Examination of Differentially Expressed Proteins at the Transcriptional Level Using qRT–PCR

**FIGURE 6:**
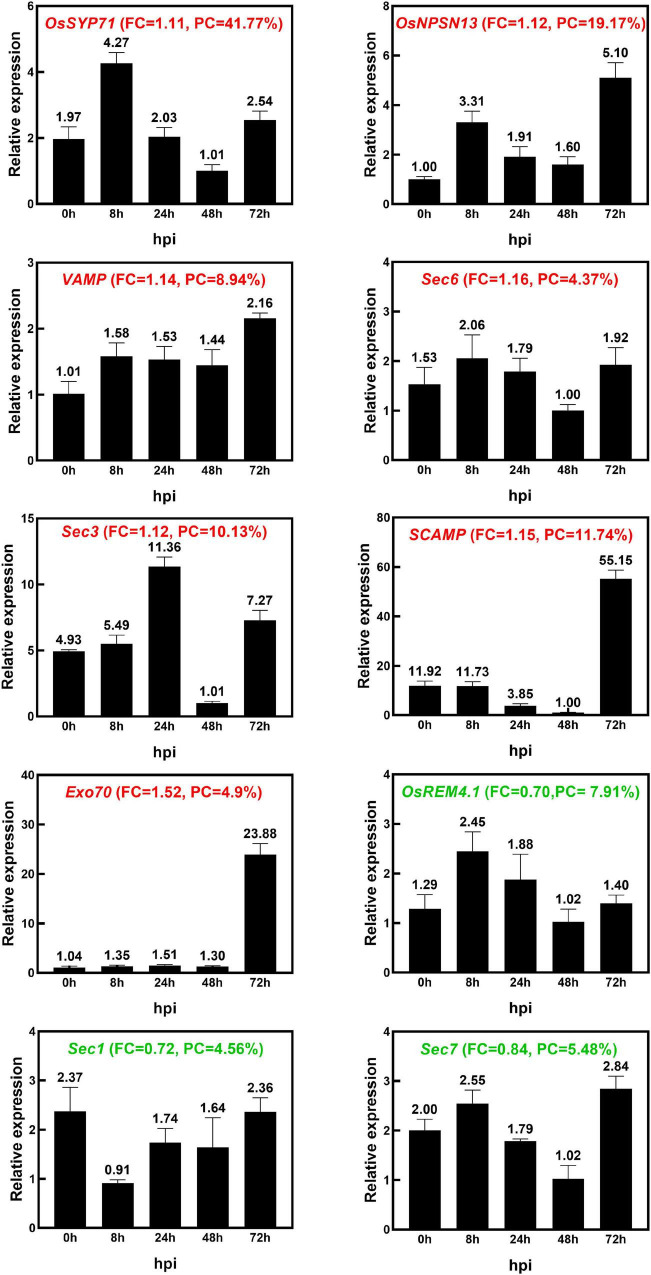
The identification of transcriptional expression patterns of 10 DEPs after *M. oryzae* infection. The expression patterns of 10 selected DEPs in Heikezijing (Hei) inoculated with the *M. oryzae* strain *Hoku1* were investigated *via* qRT–PCR. Hei seedlings were collected 0, 8, 24, 48, and 72 h after inoculation. The rice actin gene was used as an internal control. The bar chart shows the average ± standard deviation (*n* = 3). The information of Fold Change (FC) and Peptide Coverage (PC) in proteomic data was also shown in the figure. Red and green colored names represent upregulated and downregulated, respectively, in the proteomic analysis.

We examined the transcriptional pattern of ten selected DEPs using qRT–PCR. Except for OsRem4.1, the 10 DEPs were selected because they are typically related to vesicle trafficking; OsRem4.1 was chosen because it is typically located at the PM, and it has been reported that in *Arabidopsis*, Rem1.2 is related to plant immunity (Huang et al., 2019). The expression pattern analysis included seven genes that encoded upregulated proteins, including a Qc-SNARE protein (*OsSYP71*), a novel plant-specific SNARE protein (*OsNPSN13*), a vesicle-associated membrane protein (*VAMP*), an exocyst complex component protein (*Sec6*), a Sec3-PIP2 bind domain-containing protein (*Sec3*), a secretory carrier-associated membrane protein (*SCAMP*) and an exocyst subunit Exo70 family protein (*Exo70*), as well as three genes that encoded downregulated proteins, including Remorin (*OsRem4.1*), *Sec1* and *Sec7*. The transcriptional levels of *OsSYP71*, *OsNPSN13*, *VAMP*, *Sec6*, *Sec7*, *OsRem4.1*, *Sec3*, *SCAMP*, and *Exo70* were upregulated at 8 h, 24 h and 72 h after *M. oryzae* infection, while *Sec1* was downregulated at 8 h (Figure 6). *OsSYP71* and *OsNPSN13* were upregulated at 8 h, *Sec3* was upregulated at 24 h, and *SCAMP* and *Exo70* were upregulated at 72 h. According to the iTRAQ results, the transcriptional trend of *OsRem4.1* and *Sec7* was reversed, and some factors, such as mRNA splicing, translation and posttranslational regulation, as well as protein degradation, all influenced the correlation between the transcriptome and proteome data (Wei et al., 2009; Chen et al., 2015; Su et al., 2016). Additionally, to test the accuracy of the proteomic data, we identified DEP transcriptional patterns that changed slightly (fold change between 0.8 and 0.909 or 1.1 and 1.2), and the results showed that 9 of 16 proteins had comparatively good consistency (Supplementary Figure 5). Based on the iTRAQ and qRT–PCR analysis, the upregulated protein OsNPSN13 was selected for further functional analysis in resistance to rice blast.

### OsNPSN13 Is Involved in the Resistance Response Against *Magnaporthe oryzae*

**FIGURE 7:**
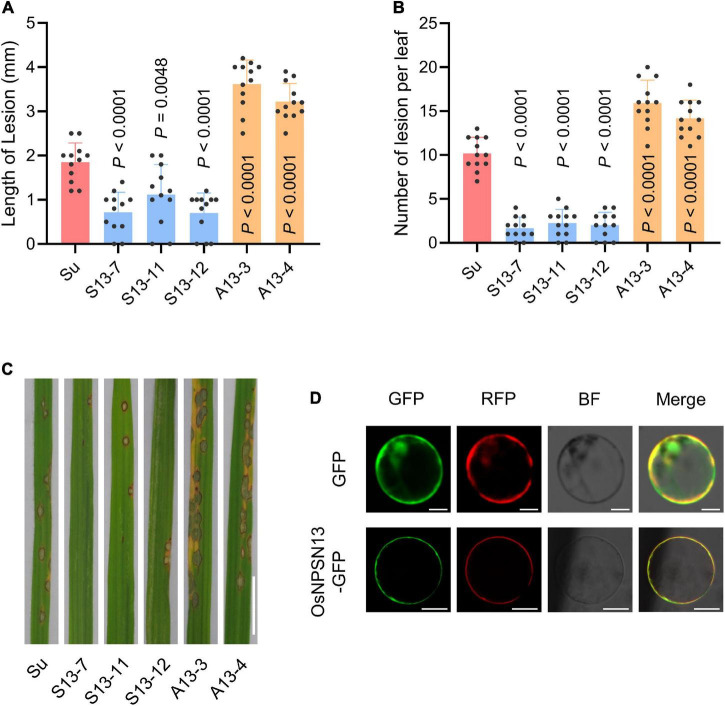
Resistance phenotype of representative transgenic plants expressing *OsNPSN13* and the subcellular localization of OsNPSN13. **(A)** Lesion length in wild-type Su and transgenic plants expressing *OsNPSN13* at 7 dpi with *M. oryzae*. **(B)** Lesion number per leaf in wild-type Su and transgenic plants expressing *OsNPSN13* inoculated with *M. oryzae* at 7 dpi. S13, transgenic overexpression lines; A13, transgenic knockdown lines. The bar charts show the average, standard deviation (*n* = 12) and *p*-value. **(C)** Rice blast resistance phenotypes of transgenic plants and wild-type Su inoculated with M. oryzae strain Hoku1. The leaves with lesions are shown. Bar = 1 cm. **(D)** The subcellular localization of OsNPSN13 transiently expressed in rice protoplasts. OsAMT3.2-RFP was used as PM marker. Bar = 5 μm.

Three *OsNPSN13*-overexpressing transgenic lines (S13-7, S13-11, and S13-12) and two knockdown transgenic lines (A13-3 and A13-4) in Su were obtained using the *Agrobacterium tumefaciens*-mediated method (driven by the 35S promoter). The characterization of the transgenic lines is shown in Supplementary Figure 6. After inoculation with rice blast fungus (strain *Hoku1*) at the seedling stage, three transgenic overexpression lines showed significantly enhanced resistance compared with wild-type Su, including fewer and shorter lesions, whereas the two knockdown transgenic lines were more susceptible to rice blast than was wild-type Su (Figures 7A–C). These results showed that OsNPSN13 was associated with resistance to rice blast fungus. The subcellular localization in protoplasts showed that OsNPSN13-GFP was mainly localized to the PM (Figure 7D).

## Discussion

**FIGURE 8:**
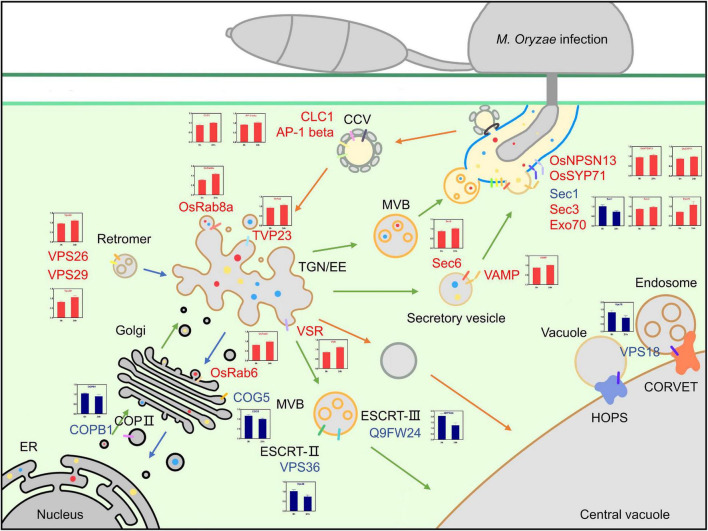
Overview of the possible vesicle trafficking network of DEPs. Possible model for the roles of DEPs in vesicle trafficking during the rice-blast fungus interaction. Upregulated DEPs are shown in red, downregulated DEPs are shown in blue, green arrows indicate exocytosis, orange arrows indicate endocytosis, and the translational patterns of DEPs are shown in the charts.

Multiple proteins associated with blast resistance in rice have been extensively studied. However, proteomic analyses of the PM protein network that participates in rice resistance against *M. oryzae* infection remain limited. In this study, a comparative iTRAQ-based proteomic analysis was performed to elucidate the changes in PM proteins during *M. oryzae* infection. A total of 831 DEPs were identified, including 434 upregulated and 397 downregulated proteins. GO enrichment analysis showed that the biological process of transport and the molecular functions associated with transporter activity were related to vesicle trafficking. KEGG analysis indicated that endocytosis and the phagosome were highly enriched pathways in the upregulated DEPs, and glutathione metabolism and glycolysis/gluconeogenesis were highly enriched pathways in the downregulated DEPs. DEPs associated with vesicle trafficking based on the GO and KEGG analyses were used to construct a protein interaction network, which showed that there were tight interactions between small GTPases and VPS proteins. A functional analysis of the DEPs yielded 20 essential proteins related to vesicle trafficking that could be mapped to a potential PM trafficking network against *M. oryzae* (Figure 8).

The exocytosis pathway is an integral component of the plant immune mechanism, and it contributes to basal resistance by transporting defense-related proteins, including PRRs; delivering antimicrobial metabolites; and mediating callose deposition (Gu et al., 2017). Five DEPs that were part of the vesicle-mediated transport term in the GO enrichment analysis, COPB1, OsRab6, COG5, OsRab8a and TVP23, were associated with protein transport from the ER to the Golgi, TGN and PM. Coatomer subunit beta COPB1 (B8BJD1) reversibly associates with Golgi non-clathrin-coated vesicles and further mediates biosynthetic protein transport from the ER *via* the Golgi to the TGN; OsRab6 (Q8H4Q9), conserved oligomeric Golgi complex subunit 5 COG5 (A2Y424) and TGN vesicle protein 23 TVP23 (Q5W723) localize to the Golgi apparatus and are involved in intra-Golgi vesicle-mediated transport and constitutive secretion (Osterrieder et al., 2009; Bottanelli et al., 2011); OsRab8a (Q84TA8) participates in vesicle trafficking between the TGN and PM to ensure the release of secretory vesicles (Huber et al., 1993; Nielsen et al., 2008). In tomato, Rab8 was identified as a potential protein that interacts with avrPto from the pathogen *Pseudomonas* sp., and this interaction possibly blocks the extracellular release of plant antimicrobial peptides (Bogdanove and Martin, 2000).

Eight DEPs, OsNPSN13, OsSYP71, VAMP, SCAMP, Sec1, Sec3, Sec6, and Exo70, were enriched in the transport term and were related to vesicle recognition and PM fusion. The Qb-SNARE protein OsNPSN13 (Q5XQQ5) localizes to the PM and is related to abiotic stress, and its expression is significantly activated in rice seedlings treated with H_2_O_2_ but is downregulated under NaCl and PEG 6000 stresses, suggesting that it might be involved in different aspects of signal transduction cascades (Bao et al., 2008a). NPSN11, a homolog of NPSN13, has been demonstrated to play an important role in disease resistance. In wheat, TaNPSN11 interacted with TaSYP132 and was distributed in vesicle structures near the cell membrane toward *Puccinia striiformis* f. sp. *tritici* (*Pst*) hypha; this behavior may play a role in vesicle-mediated resistance to stripe rust (Wang et al., 2014). ShNPSN11 in tomato is located at the PM and plays an important role in defense reactions to *O. neolycopersici* (Lian et al., 2020). In Arabidopsis, AtSYP71 belongs to the Qc-SNARE family and localizes to the PM and the ER (Suwastika et al., 2008). In rice, OsSYP71 (Q6I633) has been reported to positively contribute to rice blast resistance, and *OsSYP71* overexpression obviously enhanced resistance, including lower lesion scores and higher survival rates after *M. oryzae* inoculation (Bao et al., 2012). TaSYP71 in wheat also plays a positive role in wheat defense against *Puccinia striiformis* f. sp. *tritici* (*Pst*), and it can elevate tolerance to H_2_O_2_ and influence the expression of pathogenesis-related genes (Liu et al., 2016). However, SYP71 can also be a negative factor since it mediates the fusion of turnip mosaic potyvirus (TuMV) 6K2 vesicles with host chloroplasts during TuMV infection, which contributes to the robust replication of the viral genome. Silencing the *SYP71* gene inhibits TuMV infection and arrests the formation of the chloroplast-bound 6K2 complex (Wei et al., 2013). The SYP7 family has not been identified in yeast or mammalian SNAREs, so they probably have unique functions in the plant vesicle system (Ebine et al., 2008). Although the VAMP family protein (Q2QLJ6) of the SNARE complex and secretory carrier membrane protein (SCAMP) family protein (B8AZR2) have not been precisely classified, it is clear that these two families play an important role in vesicle trafficking. Sec1 (Q6K7L5) is involved in vesicle docking in exocytosis and contributes to membrane fusion by interacting with SNAREs or nascent trans-SNARE complexes (Südhof and Rothman, 2009; Karnahl et al., 2018). The exocyst complex (including SEC3, SEC5, SEC6, SEC8, SEC10, SEC15, EXO70 and EXO84) tethers secretory vesicles to specific sites on the PM and facilitates exocytosis (He and Guo, 2009). The exocyst subunit Sec3 (B8AMN4) belongs to the SEC3 family and plays a pivotal role in exocytosis and pollen tube growth (Bloch et al., 2016); it directly interacts with Sso1/2, facilitates the assembly of the Sso-Sec9 t-SNARE complex and stimulates membrane fusion in yeast (Yue et al., 2017). Exocyst subunit Sec6 (A2X9M7) belongs to the SEC6 family and plays a role in polar auxin transport in *Arabidopsis* (Tan et al., 2016). Exocyst subunit Exo70 (B9FW54) functions together with Sec3 in targeting the exocyst to the PM (He et al., 2007). Exocyst complexes, including Sec3, Sec6, and Exo70, may mediate precise recognition and tethering between vesicles and PM. Subsequently, Sec1, OsNPSN13, OsSYP71, VAMP, and SCAMP contribute to membrane fusion and release antimicrobial compounds and defense-related proteins to fight *M. oryzae* infection.

The endocytosis of PM-localized PRRs and LRR-receptor-like proteins (LRR-RLPs) is required for the proper dynamic localization of activated and inactivated PRRs to regulate the immune signaling response (Wang et al., 2015). Endocytic trafficking is initiated by vesicle formation and fusion and subsequently routes to endosomes and lytic vacuoles for degradation or recycling back to the PM. The major route of endocytosis is clathrin-mediated endocytosis (CME), and the cargo and vesicle coats are recruited into clathrin-coated vesicles (CCVs) and transported from the PM to early endosomes. Clathrin-coated vesicles contain two layers outside of the membrane, including adaptor proteins (APs) as the inner layer and clathrin as the outer layer. Eight DEPs, including AP-1 beta, CLC1, VPS18, VPS26, VPS29, VPS36, VSR, and Q9FW24, were included in the endocytosis and transport terms in the KEGG and GO enrichment analyses, respectively. AP-1 complex subunit beta (Q0DRT9) mediates TGN-endosome and Golgi-vacuole intracellular protein transport (Zwiewka et al., 2011; Feng et al., 2017); Clathrin light chain 1 CLC1 (Q7XKE9) combines with clathrin heavy chain (CHC) and forms the outer layer of clathrin-coated vesicles (CCVs). We hypothesize that the activated PRRs at the PM are essential for the continuation of immune signaling, so the downregulated proteins can lead to fewer activated PRRs being recycled and degraded in the vacuole.

After transport from the PM to the TGN/EE by clathrin-coated vesicles, the recycled proteins are sorted into different locations, including to the vacuole for degradation or recruitment to the PM. Vacuolar protein sorting-associated proteins (VPSs) play an essential role in this process. Two tethering complexes, homotypic fusion and protein sorting (HOPS) and class C core vacuole/endosome tethering (CORVET), mediate transport from endosomes to vacuoles and share mutual core proteins, including VPS11, VPS18 (Q6ZKF1), and VPS16 (Vukašinovic and Žárský, 2016; Takemoto et al., 2018). VPS36 (B8AB94) is a component of endosomal sorting complexes required for transport (ESCRT)-II, which has ubiquitin-binding activity and is critical for MVB formation and vacuolar biogenesis to degrade proteins (Teo et al., 2004; Hsua and Jauh, 2017; Wang et al., 2017). VPS36 has been reported to be associated with the transport of PIN1, AUX1, and PIP1 family members and with root gratitropism in *Arabidopsis*, but its function in plant immunity remains unknown. The SNF7 domain-containing protein (Q9FW24) is the core component of ESCRT-III that mediates MVB biogenesis by facilitating membrane bending, scission and fusion (Ibl et al., 2012). Vacuolar-sorting receptor VSR (Q0JA85) mediates Golgi-to-vacuole transport. VPS18, VPS36, and Q9FW24 were downregulated, and this result suggested that the degradation of some specific proteins may be restricted during *M. oryzae* infection, but the details are still elusive and require further investigation.

In addition to being degraded by vacuoles, some proteins may be recruited back to the PM by retromers to perform their function. It has been reported that flg22-activated FLS2 is recruited to the extrahaustorial membrane (EHM) induced by *Phytophthora infestans* (Bozkurt et al., 2015). The retromer is a conserved complex located on the cytosolic face of endosomes and VPS26 (Q2R0X0), VPS29 (Q6ETY0) and VPS35; it regulates the retrieval of cargo from the endocytic system to the TGN or PM (Zelazny et al., 2013; Priya et al., 2015). VPS26 and VPS29 were upregulated, suggesting that retrograde transport was enhanced and potentially included the recruitment of defense-associated proteins to the PM. The roles of receptor-mediated endocytosis may be complicated in plants; however, our current knowledge about its roles in regulating the activity of immunity-related proteins remains limited. Many DEPs associated with endocytosis were found in this study, which provides a direction for future research efforts.

An overview of the potential vesicle trafficking response to *M. oryzae* infection is shown in Figure 8. In exocytosis, COPB1, COG5, OsRab6, OsRab8a, and TVP23 mediate the transport of synthesized proteins from the ER to the Golgi and TGN/EE, assembly in vesicles and movement toward the PM; Sec1, Sec3, Sec6, and Exo70 ensure recognition and tethering between vesicles and the PM; and membrane fusion is completed by VAMP, OsNPSN13, and OsSYP71. In endocytosis, the formation and assembly of clathrin-coated vesicles require AP-1 beta and CLC1; the sorting of recycled proteins and vacuole-targeted degradation are mediated by VPS18, VPS36, VSR and Q9FW24; and the recruitment of proteins to the Golgi or PM is completed by retromers, including VPS26 and VPS29.

After thoroughly exploring the iTRAQ data *via* the functional analysis of DEPs and identification *via* qRT–PCR, we ultimately focused on the significantly changed SNARE protein OsNPSN13. Overexpressing OsNPSN13 in rice led to enhanced resistance, while knocking down OsNPSN13 expression led to serious susceptibility. SNARE proteins are related to the secretory transport of PRRs and PR proteins to the PM (Beck et al., 2012). It has been reported that in wheat, knocking down TaNPSN13 reduced ROS-mediated plant defense at stripe rust infection sites, indicating that it might be involved in the delivery of ROS-related materials; in addition, TaNPSN11 and its structural homologs mediated the vesicle trafficking of plant defense-related materials between the Golgi apparatus and the PM based on the bubbly layered structures in immunolocalization assays (Wang et al., 2014). In rice, OsNPSN13 has been related to H_2_O_2_ stress, and in this study, it was mainly located at the PM, with a small portion in the cytoplasm, and it showed resistance against *M. oryzae*. We hypothesize that OsNPSN13 may have a dual function in transporting both ROS and defense-related proteins to the PM by forming different SNARE complexes in different vesicles. Our study provides initial evidence that OsNPSN13 is involved in vesicle-mediated plant immunity, but the detailed molecular mechanism requires further investigation.

The PM proteomic analysis presented an overview of the protein expression profile in resistant rice in response to *M. oryzae* infection at 24 h. The iTRAQ data presented here will help us to further understand the molecular mechanisms of resistance to rice blast. Many DEPs were involved in transport, phagosome and endocytosis. The protein interaction network showed higher degrees of interaction among multiple Ras-related proteins, including OsRab6, OsRab8a, GTP1, OsRIC1, VPS18, VPS26, VPS29, TuBB1, TuBB6 and serine/threonine phosphatases. qRT–PCR proved the accuracy of the iTRAQ data at the transcriptional level. OsNPSN13 was upregulated during *M. oryzae* infection, and the identification of overexpression and knockdown mutants showed that it indeed played a role in the process of rice blast resistance. Our study uncovered a complex network that associates the PM (especially membrane trafficking) with rice blast resistance. In the future, studies on the functions of the specific proteins found in this study will help to explore the mechanisms of resistance against *M. oryzae* infection.

## Data Availability Statement

The datasets presented in this study can be found in online repository. The name of the repository is PRIDE proteomics repository, and the accession number is PXD028545.

## Author Contributions

YB and ZZ conceived of the presented idea and drafted the manuscript with input from all authors. YB, ZZ, ML, HeZ, YY, LM, WW, YF, NH, XW, KL, SD, and HT implemented, collected, and analyzed the data. YB, ZZ, LM, HeZ, and YY conducted most experiments. YB was involved in the planning and supervision of the work. All authors contributed to the article and approved the submitted version.

## Conflict of Interest

The authors declare that the research was conducted in the absence of any commercial or financial relationships that could be construed as a potential conflict of interest.

## Publisher’s Note

All claims expressed in this article are solely those of the authors and do not necessarily represent those of their affiliated organizations, or those of the publisher, the editors and the reviewers. Any product that may be evaluated in this article, or claim that may be made by its manufacturer, is not guaranteed or endorsed by the publisher.

## Footnotes

1
http://geneontology.org/

2
https://www.genome.jp/kegg/

3
https://string-db.org
