# Supplementary material for: Comparative Proteomic Analysis of Plasma Membrane Proteins in Rice Leaves Reveals a Vesicle Trafficking Network in Plant Immunity That Is Provoked by Blast Fungi
Source: Front Plant Sci. 2022 Apr 25;13:853195. doi: 10.3389/fpls.2022.853195 (PMC9083198; doi:10.3389/fpls.2022.853195)
Supplement: Supplementary file 10 [file Presentation_1.PPTX]

## Slide 1
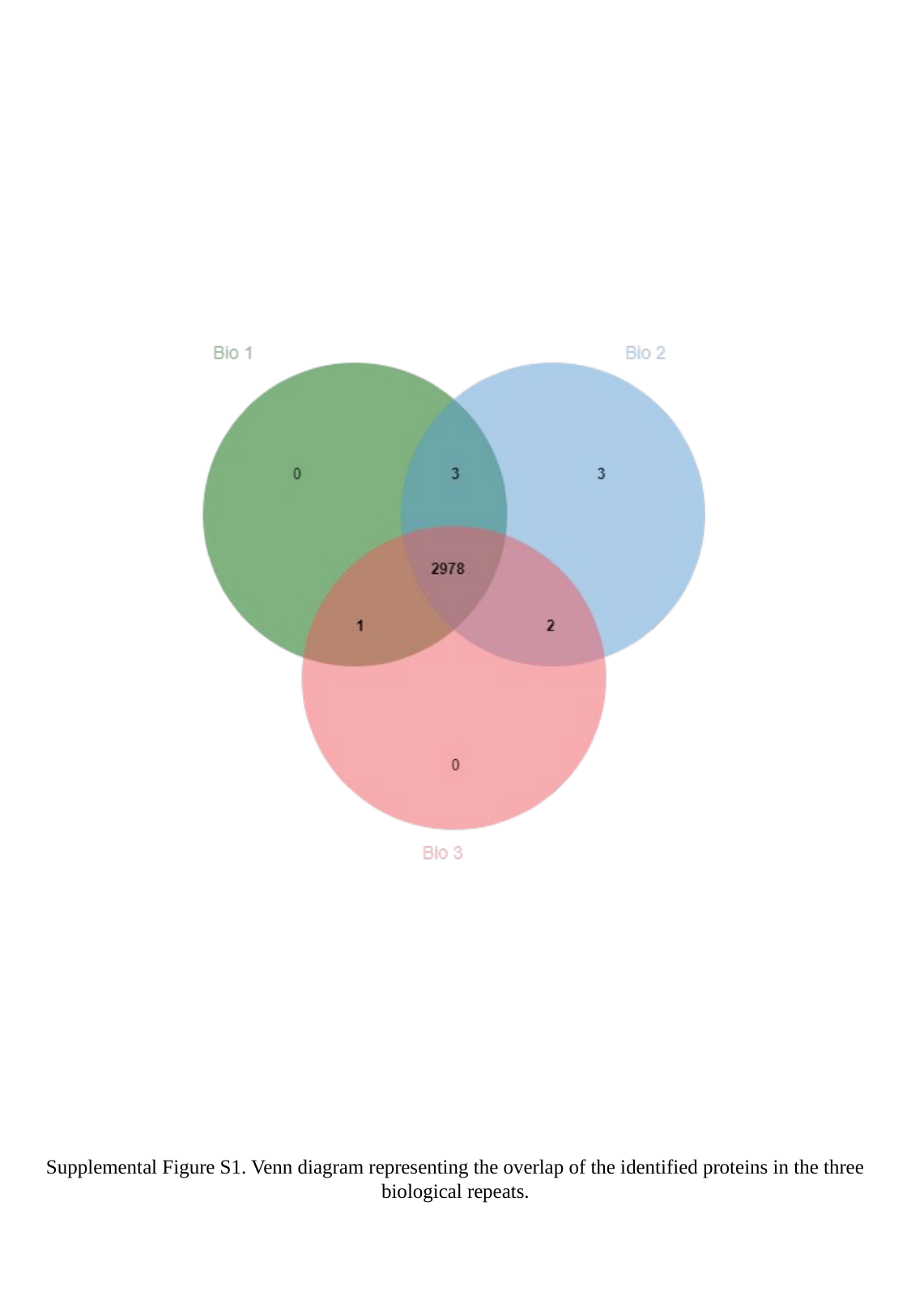

Supplemental Figure S1. Venn diagram representing the overlap of the identified proteins in the three biological repeats.

## Slide 2
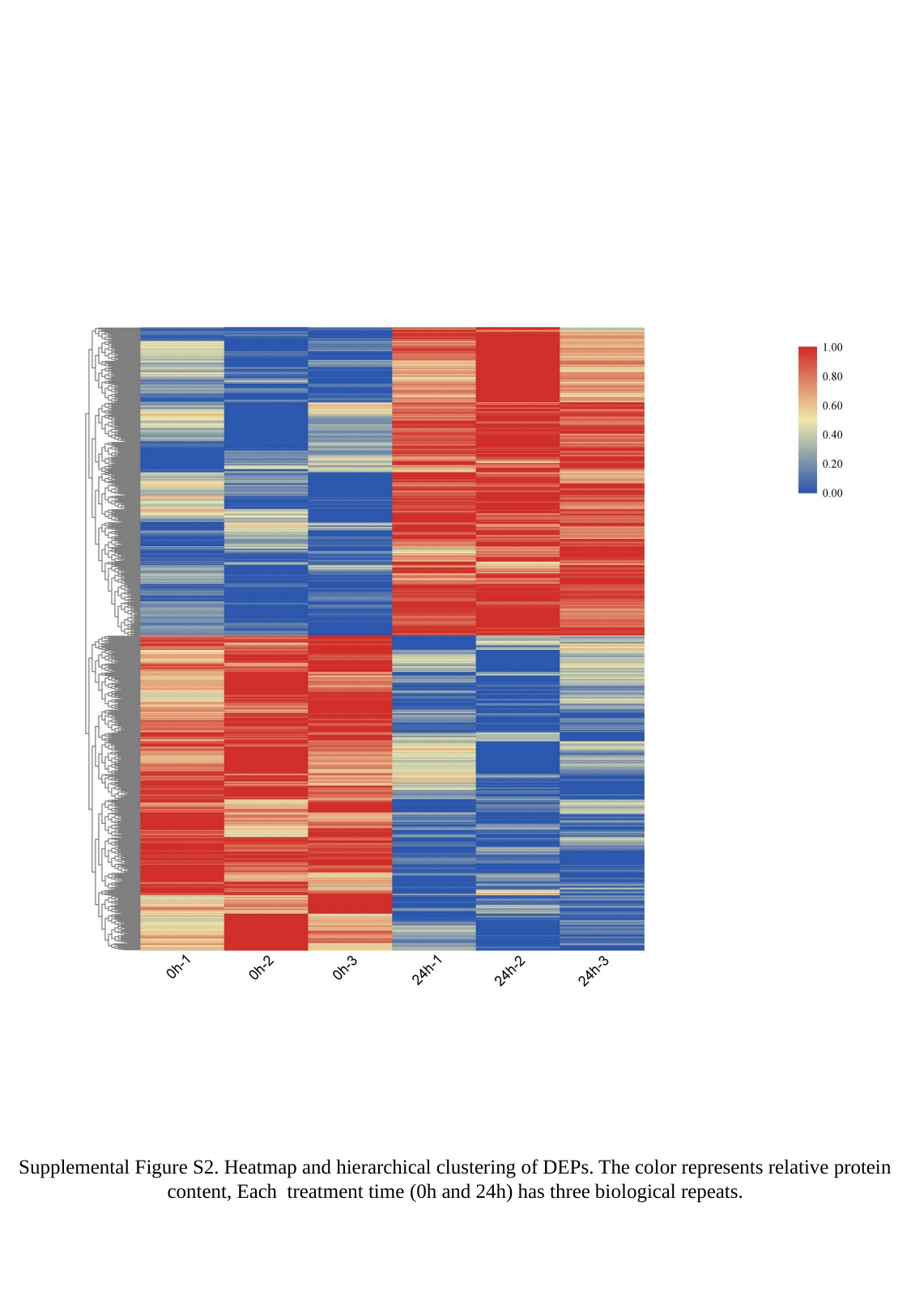

Supplemental Figure S2. Heatmap and hierarchical clustering of DEPs. The color represents relative protein content, Each treatment time (0h and 24h) has three biological repeats.

## Slide 3
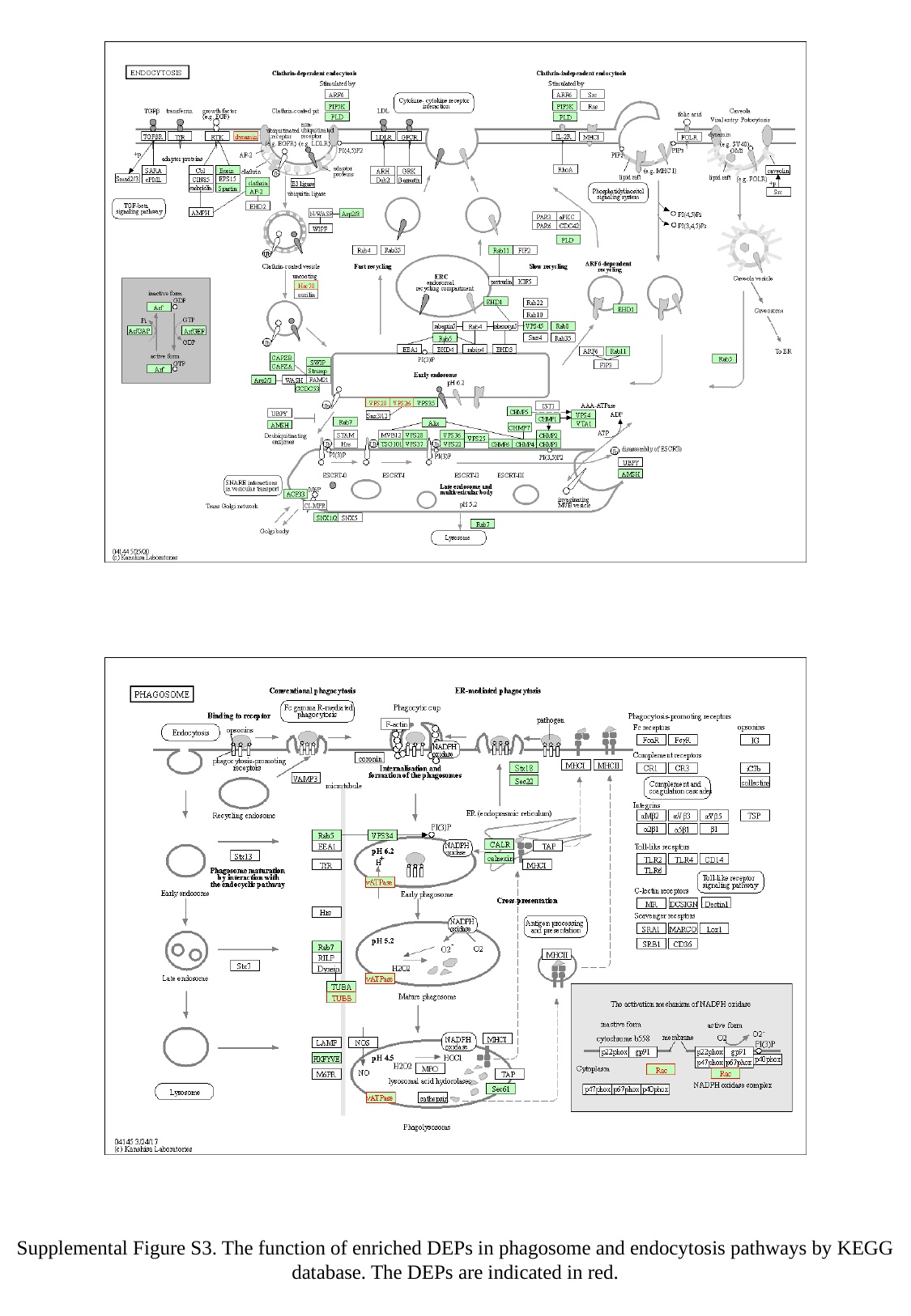

Supplemental Figure S3. The function of enriched DEPs in phagosome and endocytosis pathways by KEGG database. The DEPs are indicated in red.

## Slide 4
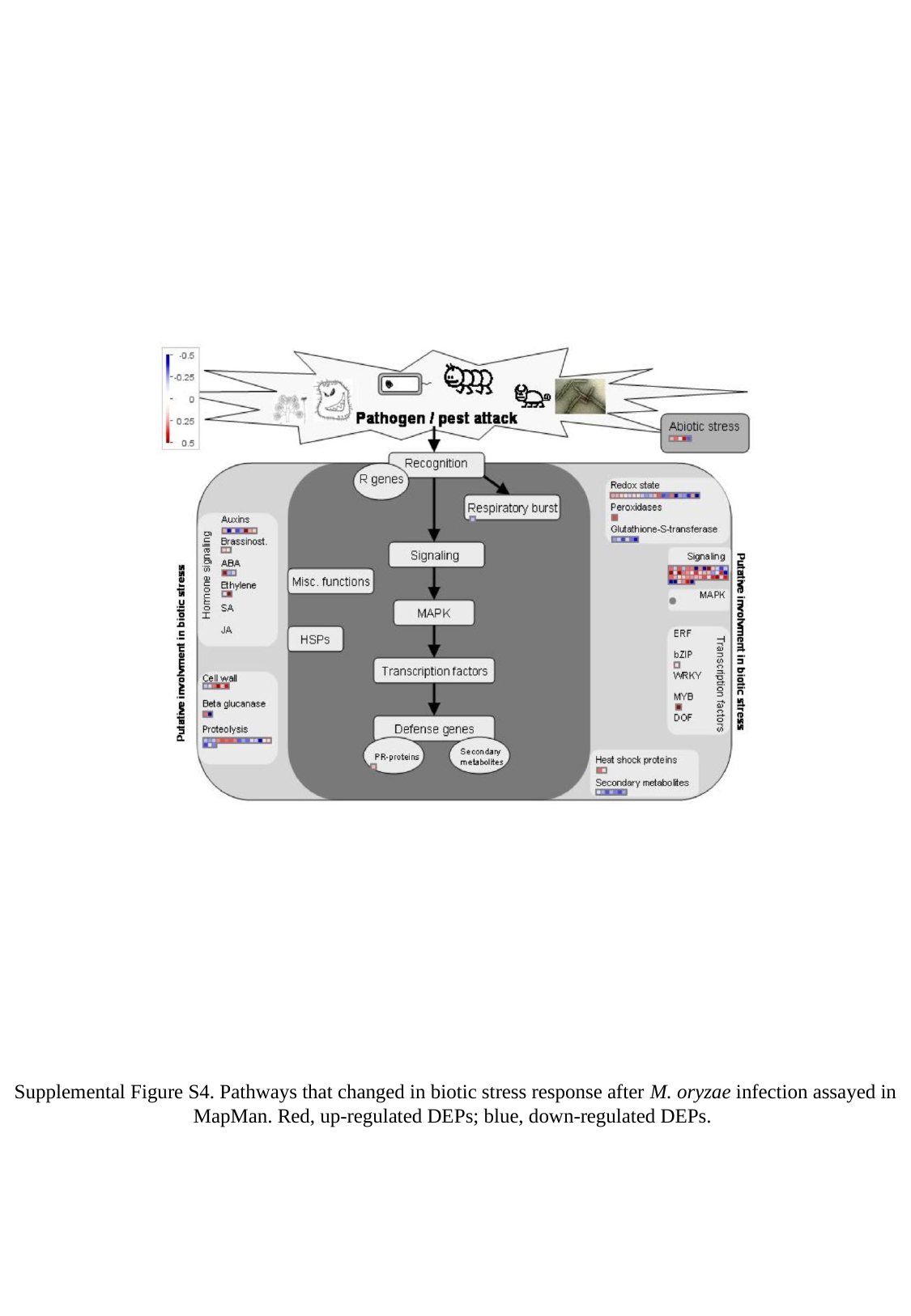

Supplemental Figure S4. Pathways that changed in biotic stress response after M. oryzae infection assayed in MapMan. Red, up-regulated DEPs; blue, down-regulated DEPs.

## Slide 5
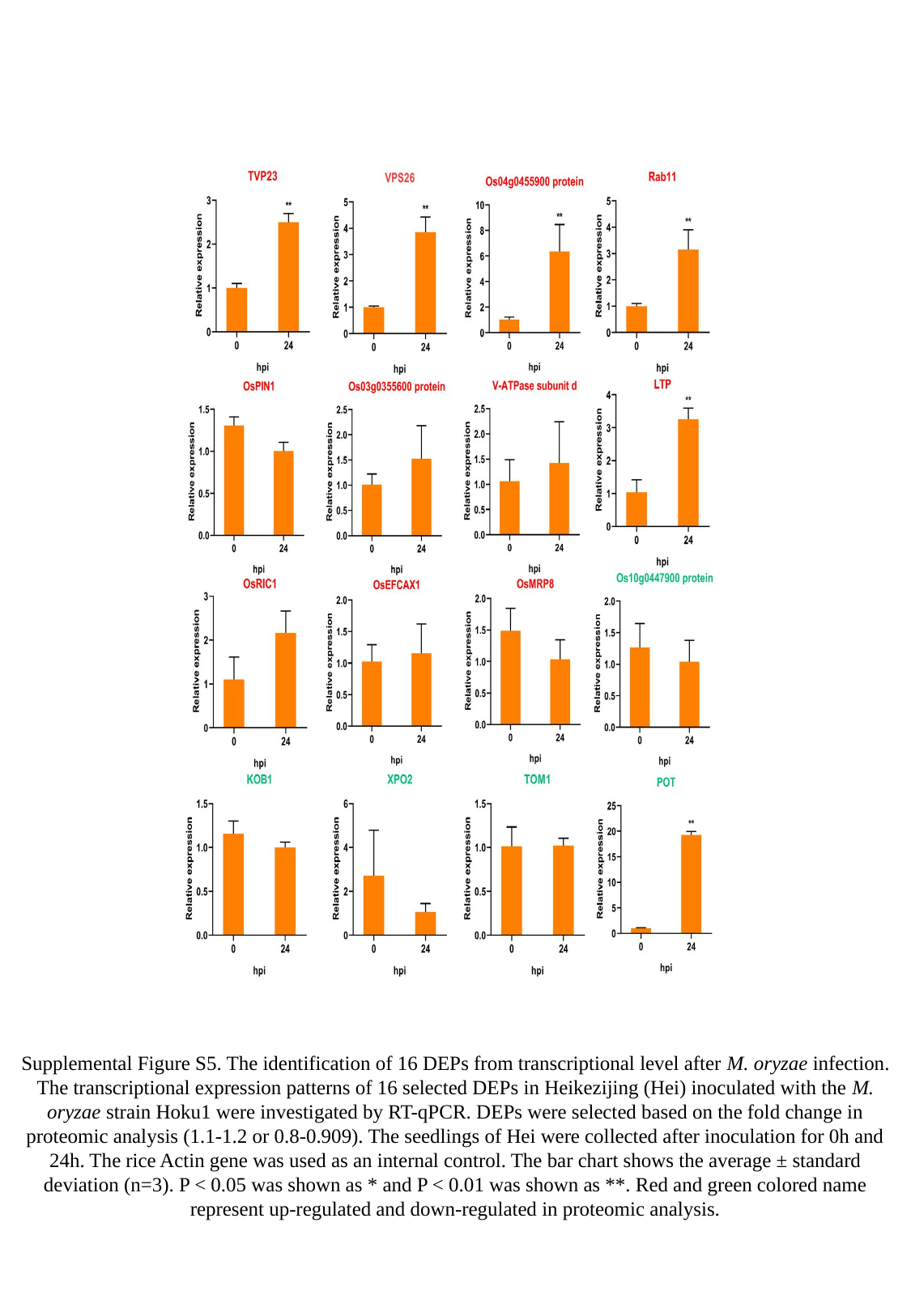

Supplemental Figure S5. The identification of 16 DEPs from transcriptional level after M. oryzae infection. The transcriptional expression patterns of 16 selected DEPs in Heikezijing (Hei) inoculated with the M. oryzae strain Hoku1 were investigated by RT-qPCR. DEPs were selected based on the fold change in proteomic analysis (1.1-1.2 or 0.8-0.909). The seedlings of Hei were collected after inoculation for 0h and 24h. The rice Actin gene was used as an internal control. The bar chart shows the average ± standard deviation (n=3). P < 0.05 was shown as * and P < 0.01 was shown as **. Red and green colored name represent up-regulated and down-regulated in proteomic analysis.

## Slide 6
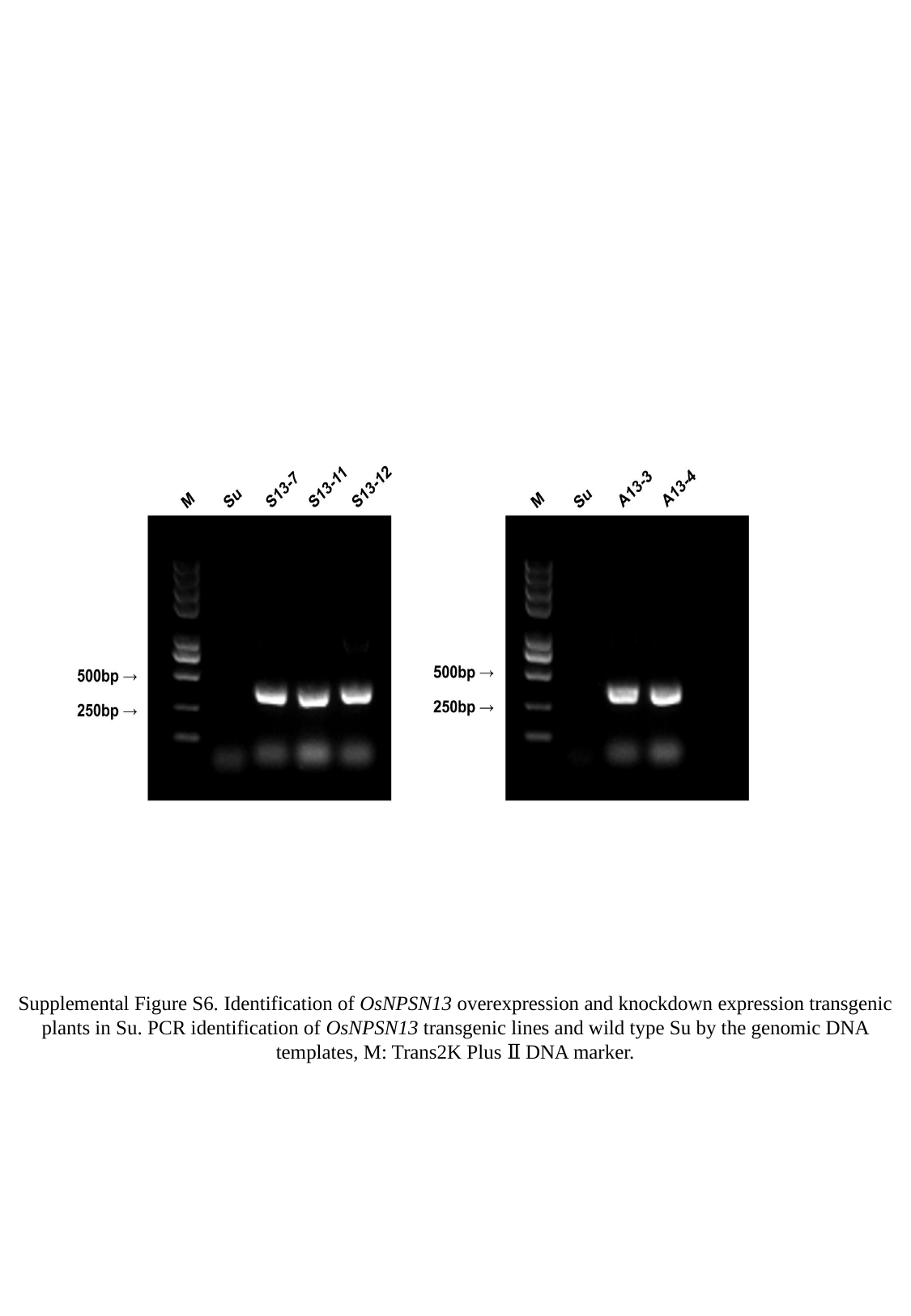

Supplemental Figure S6. Identification of OsNPSN13 overexpression and knockdown expression transgenic plants in Su. PCR identification of OsNPSN13 transgenic lines and wild type Su by the genomic DNA templates, M: Trans2K Plus Ⅱ DNA marker.
